# Strategies to reduce clinical inertia in hypertensive kidney transplant recipients

**DOI:** 10.1186/1471-2369-8-10

**Published:** 2007-07-27

**Authors:** James Kiberd, Romauld Panek, Bryce Kiberd

**Affiliations:** 1 Department of Medicine, Dalhousie University, Halifax, Nova Scotia, Canada

## Abstract

**Background:**

Many kidney transplant recipients have hypertension. Elevated systolic blood pressures are associated with lower patient and kidney allograft survival.

**Methods:**

This retrospective analysis examined the prevalence of clinical inertia (failure to initiate or increase therapy) in the treatment of hypertension before and after the introduction of an automated device (BpTRU) in the kidney transplant clinic.

**Results:**

Historically only 36% (49/134) of patients were prescribed a change in therapy despite a systolic blood pressure ≥ 130 mmHg. After the introduction of BpTRU, 56% (62/110) of the patients had a change in therapy. In a multivariate logistic regression analysis of the entire cohort (n = 244) therapeutic changes were associated with higher blood pressures (OR 1.08 per mmHg, 95% CI 1.04–1.12) and use of the BpTRU (OR 2.12, 95% CI 1.72–3.83). In addition patients on more medications were also more likely to have a change in therapy.

**Conclusion:**

Blood pressure measurement with automated devices may help reduce clinical inertia in the kidney transplant clinic.

## Background

Several studies have shown that higher blood pressures are associated with reduced kidney allograft and patient survival [1-3]. For every 10 mmHg higher systolic pressure graft loss is increased by 12–15% [2,3]. The guidelines recommend blood pressure targets of <130/85 mmHg [4]. Despite these guidelines many patients have blood pressures above this level [1,3].

Blood pressure is difficult to control in kidney transplant recipients in part because of the associated immunosuppressive medication, impaired graft function, older age, obesity and diabetes mellitus [3]. Many studies however report that clinicians under treat patients [5-7]. The literature describes the phenomena of clinical inertia when physicians fail to initiate or intensify therapy when warranted [6,7]. There is a growing literature on clinical inertia and hypertension in the general population but less is known in the kidney transplant population.

In a recent American Heart Association scientific statement, the authors point out the shortcomings of auscultatory office blood pressures in the clinical practice despite their long standing importance in clinical research [8]. Patients commonly report that their blood pressures are better at home and are aware of the white coat phenomena. Many clinics are not performing research quality blood pressure measurements. For these reasons clinicians may doubt the precision or certainty of the measurement and a therapeutic change will be deferred. Ambulatory blood pressure monitoring devices are one strategy to increase diagnostic accuracy and may better predict outcomes [8]. Unfortunately 24 hour ambulatory blood pressure monitoring is not convenient, especially for patients traveling great distances. One automated device that meets hypertension standards and that can be used in the office is the BpTRU automated device [9,10]. The mean difference between the reference standard systolic and BpTRU systolic measurement in one study was 0.47 ± 5.4 mmHg with 89.2 % of the measurements within 5 mmHg [10]. The BpTRU was also a better estimate of ambulatory blood pressures than routine office measurements [11] We introduced the BpTRU device in our clinic on the recommendation of our hospital hypertension experts. In this report we describe our experience with the device and explore the impact on therapeutic changes in hypertensive kidney transplant recipients.

## Methods

In October 2005, the clinic introduced the BpTRU device (VSM Medtech Ltd, Coquitlam, British Columbia, Canada). This device employs the oscillometric technique used by most home and ambulatory blood pressure devices. It reads 6 blood pressure measurements every 2 minutes while the patient is seated quietly alone in a room. The first measurement is ignored and the last 5 measurements are averaged.

In November the clinic prospectively used the device on patients with a blood pressure ≥ 130 mmHg systolic by the clinic's aneroid syhygmomanometer. Both blood pressure values were recorded. To test the hypothesis that the device had an impact on initiating or intensifying therapy, these patients (BpTRU group) were retrospectively reviewed. The comparators were historic patients (Control group) with a systolic blood pressure ≥ 130 mm Hg who had been in clinic within the year prior to the introduction of the BpTRU. Inclusion into both cohorts was based on the clinic's aneroid syhygmomanometer blood pressure. This was a retrospective analysis and individual informed consent was not obtained. Permission to perform the study was provided by our institution research ethics committee.

All patients were adults (>18) and were either kidney alone or combined kidney pancreas transplant recipients. Patients transplanted within 6 months were excluded. For patients who had several BpTRU measurements only the first was analyzed. Data on patient age, weight, height, sex, serum creatinine, glomerular filtration rate, diabetes mellitus status, resistant hypertension, attending physician, antihypertensive and immunosuppression medication use were collected. Glomerular filtration rate was estimated by the MDRD 4 variable equation [12]. Resistant hypertension was defined as receiving 3 or more blood pressure lowering medications with one being a diuretic [13]. The outcome measure was a change in therapy. For the purpose of this study newly added antihypertensive therapy, increase in the dose of current antihypertensive therapy, and reductions in calcineurin inhibitor or prednisone doses were consider therapeutic changes. Our clinic's written policy is that blood pressure be controlled to <130/85 mmHg [4]. Patients had already been given dietary advice to achieve and maintain ideal body weight and limit salt intake. No other specific policies or procedures were introduced over the study time period.

A statistically significant increase in therapy with the introduction of BpTRU was tested by Chi-square analysis. Differences between group (Control versus BpTRU) demographics were tested by Chi-square for dichotomous variables and ANOVA for continuous variables. A level of significance was set at 5%. A multivariate logistic regression analysis was performed on the entire cohort (n = 244) to look at independent predictors of a therapeutic change.

## Results

Table 1 shows the patient demographics and clinical descriptors of the two groups. There were slightly more patients with diabetes mellitus in the control population whereas diastolic blood pressure was higher in the BpTRU sample. There were no statistical differences between individual antihypertensive medications such as calcium channel blockers, beta blockers, angiotensin enzyme inhibitors, angiotensin receptor blockers, diuretics or other blood pressure medication. Calcineurin inhibitor and prednisone use were similar.

**Table 1:** Demographic and clinical descriptors

	Control N = 134	BpTRU N = 110	prob
Age (years)	52 ± 13	53 ± 13	
Sex (male)	83 (62)	66 (60)	
Diabetes mellitus (%)	54 (40)	30 (27)	0.042
ESRD n (%)			
Diabetes mellitus	27 (20)	20 (18)	
Glomerulonephritis	23 (17)	22 (20)	
Polycystic kidney disease	46 (34)	36 (33)	
Hypertension	16 (12)	14 (13)	
Interstitial nephritis	13 (10)	12 (11)	
Other	9 (7)	6 (5)	
Duration of transplant (yrs)	7.7 ± 6.1	7.2 ± 6.1	
Body weight (kg)	85.5 ± 19.1	83.1 ± 19.2	
BMI (kg/m^2^)	30.1 ± 6.7	28.9 ± 6.1	
Creatinine (μmol/L)	141 ± 52	152 ± 72	
GFR ml/min/1.73 m^2^	65.9 ± 26.0	61.3 ± 28.3	
BP systolic (mm Hg)	143 ± 10	145 ± 7	0.082
BP diastolic (mm Hg)	81 ± 10	85 ± 11	0.002
BpTRU systolic	NA	136 ± 18	
BpTRU diastolic	NA	83 ± 13	
Change made (%)	49 (36)	62 (56)	0.002
CNI (yes) (%)	124 (93)	99 (90)	
Prednisone (yes) (%)	102 (76)	87 (79)	
AHM total meds	2. 2 ± 1.1	2.2 ± 1.2	
Resistant HTN	39 (29%)	35 (32%)	

BMI- Body mass index (kg/m^2^)

GFR- glomerular filtration rate (MDRD formula)

CNI- calcineurin inhibitor

AHM- antihypertensive medication

HTN- hypertension

There were more changes in therapy (p = 0.002) in the BpTRU group (56%) compared to historic controls (36%) (Table 1). Changes in therapy included new antihypertensive medication (n = 18), increase in antihypertensive medication dose (n = 22) and lowered immunosuppression (n = 9) in the control group. The medications added included ACEI/ARB (n = 9), diuretic (n = 3), calcium channel blocker (n = 3) beta-blocker (n = 2), and alpha-blocker (n = 1). For the BpTRU group changes in therapy included new antihypertensive medication (n = 25), increase in antihypertensive medication dose (n = 23) and lowered immunosuppression (n = 14). The medications added included ACE/ARB (n = 13), diuretic (n = 8), calcium channel blocker (n = 1) and beta-blocker (n = 3). The types of changes made were not statistically different.

Analysis of the entire cohort by logistic regression analysis (Table 2) found that level of systolic blood pressure, number of antihypertensive medications the patient was already on, and use of the BpTRU device were associated with more changes in therapy. Other variables such as age, weight, GFR, BMI, attending physician, kidney function, individual antihypertensive or immunosuppressive medication use were not significant predictors.

**Table 2:** Associations with therapeutic changes: multivariate logistic regression analysis

	EXP(B)	95% CI	probability
BP systolic (per mm Hg)	1.079	1.041–1.119	<0.001
BpTRU (yes)	2.118	1.717–3.833	0.013
AHM total (per med)	1.417	1.085–1.851	0.011

AHM -antihypertensive medication

Adjusted for patient age, sex, BMI, physician, and diabetes mellitus status

Within the BpTRU group not all patients were hypertensive by the automated device. Only 72 of the 110 patients (66%) had a blood pressure that was ≥ 130 mmHg systolic with the BpTRU. Therapeutic changes were made in 49 of the 72 (68%). In those with BpTRU <130 mmHg systolic therapeutic changes were made in 13 of the 38 (38%) patients.

## Discussion

The results show that more significantly therapeutic changes were made in hypertensive kidney transplant recipients being followed in the clinic during the period of BpTRU use. The device may have provided some measurement certainty to both patient and physician to the extent that more changes were made. The two other factors associated with therapeutic changes also provide measures of certainty. Patients on more medications may be perceived by the clinician as more likely to have uncontrolled blood pressure. Similarly patients with higher blood pressures are more likely to have truly elevated pressures.

Clinical inertia in the treatment of hypertension is not a new phenomenon but has not been reported in the kidney transplant literature. In a recent study of outpatient general population practices in the southeastern United States, 7253 hypertensive patients were evaluated [5]. Changes in therapy occurred in only 13.1% of clinic visits in patients with blood pressures exceeding >140/90 mmHg. The authors estimated that increasing medication doses in 30% of patients would have increased the number of patients in target from 45.1% to 65.9%. In our historic group of kidney transplant recipients changes were made in 36% of patients. Although this might appear to be significantly better, therapeutic changes were made in 49% of hypertensive kidney disease patients attending Canadian general nephrology clinics [14]. Although differences in the study populations preclude detailed comparisons, therapeutic changes are occurring in a <50% of patients even under specialist care. If antihypertensive therapy is not intensified in the kidney transplant population it should be no surprise that most will have blood pressures exceeding target levels [1,3].

There are several limitations of the study. This study was not randomized, nor blinded. We also cannot say that other secular influences might have also been associated with changes in prescribing patterns. We used historic rather than concurrent controls. Follow up blood pressures were not obtained. However, more than two thirds of patients in both groups did not meet the criteria for hypertension resistance, suggesting more intense therapy should be helpful. Our clinic blood pressures were best described as casual. Our clinic does not ensure the research standard for a clinical trail (no caffeine or nicotine 20 minutes before, seated with back supported for 5 minutes, not engaged in conversation, two measurements a few minutes apart, agreement within 5 mm Hg, avoid terminal digit preference) [15]. Clinics that ensure this level of quality probably do not require this type of assistance to improve blood pressure certainty. We analysed systolic blood pressure as the primary variable rather than diastolic as this most closely correlates with outcome [1-3] Ambulatory blood pressures would be preferred and are carried out at our center, but geographical issues and availability limit this method in many of our patients. We unfortunately did not collect data on patient home monitoring. In addition, the device has no terminal digit preference and can be used efficiently in busy clinics.

## Conclusion

Although there are no randomized control trials in the kidney transplant population there is a wealth of cohort data and considerable evidence in the general population supporting blood pressure control to preserve kidney function and reduce mortality. It is not clear from this small single center retrospective review whether an automated device or other strategies to increase measurement certainty will reduce clinical inertia over the long run and improve overall patient outcomes. This degree of evidence would require a large randomized trial. Nonetheless we hope this study encourages others to scrutinize their practice and consider the necessary steps to ensure that blood pressure targets are achieved with the ultimate goal of increasing patient and allograft survival.

## Competing interests

The author(s) declare that they have no competing interests.

## Authors' contributions

All authors participated in the design, data collection, discussion of the results and approval of the manuscript. BK performed the statistical analysis and wrote the initial draft of the paper.

## Pre-publication history

The pre-publication history for this paper can be accessed here:



## References

[B1] Opelz G, Dohler B, Collaborative Transplant Study (2005). Improved long-term outcomes after renal transplantation associated with blood pressure control. Am J Transplant.

[B2] Mange KC, Cizman B, Joffe M, Feldman HI (2000). Arterial hypertension and renal allograft survival. JAMA.

[B3] Kasiske BL, Anjum S, Shah R (2004). Hypertension after kidney transplantation. Am J Kidney Dis.

[B4] EBPG Expert Group on Renal Transplantation (2002). European best practice guidelines for renal transplantation. Section IV: Long-term management of the transplant recipient. IV.5.2. Cardiovascular risks. Arterial hypertension. Nephrol Dial Transplant.

[B5] Okonofua EC, Simpson KN, Jesri A, Rehman SU, Durkalski VL, Egan BM (2006). Therapeutic inertia is an impediment to achieving the Healthy People 2010 blood pressure control goals. Hypertension.

[B6] O'Connor PJ (2003). Overcome clinical inertia to control systolic blood pressure. Arch Intern Med.

[B7] Fine LJ, Cutler JA (2006). Hypertension and the treating physician: understanding and reducing therapeutic inertia. Hypertension.

[B8] Pickering TG, Hall JE, Appel LJ (2005). Recommendations for blood pressure measurement in humans and experimental animals: part 1: blood pressure measurement in humans: a statement for professionals from the Subcommittee of Professional and Public Education of the American Heart Association Council on High Blood Pressure Research. Circulation.

[B9] Campbell NR, Conradson HE, Kang J, Brant R, Anderson T (2005). Automated assessment of blood pressure using BpTRU compared with assessments by a trained technician and a clinic nurse. Blood Press Monit.

[B10] Mattu GS, Heran BS, Wright JM (2004). Overall accuracy of the BpTRU – an automated electronic blood pressure device. Blood Press Monit.

[B11] Beckett L, Goodwin M (2005). The BpTRU automatic electronic blood pressure to 24 hour ambulatory blood pressure monitoring in the assessment of blood pressure in patients with hypertension. BMC Cardiovascular Disorders.

[B12] Levey AS, Greene T, Kusek JW, Beck GJ, Group MS (2000). A more accurate equation to estimate glomerular filtration rate from serum creatinine. J Am Soc Nephrol.

[B13] Chobanian AV, Bakris GL, Black HR, Cushman WC, Green LA, Izzo JL, Jones DW, Materson BJ, Oparil S, Wright JT, Roccella EJ, National Heart, Lung and Blood Institute Joint National Committee on Prevention, Detection, Evaluation, and Treatment of High Blood Pressure; National High Blood Pressure Education Program Coordinating Committee (2003). The Seventh Report of the Joint National Committee on Prevention, Detection, Evaluation and Treatment of High Blood Pressure: The JNC 7 Report. JAMA.

[B14] Tonelli M, Gill J, Pandeya S, Bohm C, Levin A, Kiberd BA (2002). Barriers to blood pressure control and angiotensin enzyme inhibitor use in Canadian patients with chronic renal insufficiency. Nephrol Dial Transplant.

[B15] Tomson CR (2003). Ambulatory blood pressure measurement in kidney transplantation: an overview. Transplantation.

